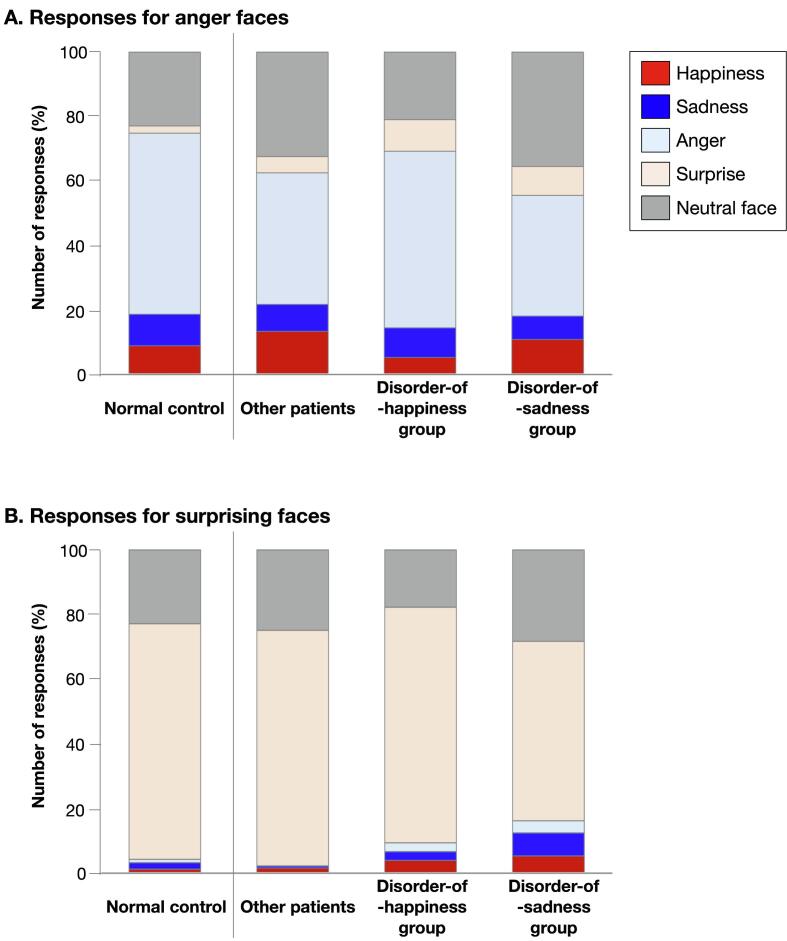# Posterior-prefrontal and medial orbitofrontal regions play crucial roles in happiness and sadness recognition

**DOI:** 10.1016/j.nicl.2022.103072

**Published:** 2022-06-02

**Authors:** Riho Nakajima, Masashi Kinoshita, Hirokazu Okita, Mitsutoshi Nakada

**Affiliations:** a Department of Occupational Therapy, Faculty of Health Science, Institute of Medical, Pharmaceutical and Health Sciences, Kanazawa University, Kanazawa, Japan; b Department of Neurosurgery, Faculty of Medicine, Institute of Medical, Pharmaceutical and Health Sciences, Kanazawa University, Kanazawa, Japan; c Department of Physical Medicine and Rehabilitation, Kanazawa University Hospital, Kanazawa, Japan

**Keywords:** Emotion recognition, Basic emotion, Happiness, Sadness, Structural connectivity

## Abstract

The core brain regions responsible for basic human emotions are not yet fully understood. We investigated the key areas responsible for emotion recognition of facial expressions of happiness and sadness using data obtained from patients who underwent local brain resection. A total of 44 patients with right cerebral hemispheric brain tumors and 33 healthy volunteers were enrolled and subjected to a facial expression recognition test. Voxel-based lesion-symptom mapping was performed to investigate the relationship between the accuracy of emotion recognition and the resected regions. Consequently, trade-off relationships were discovered: the posterior-prefrontal region was related to a low score of happiness recognition and a high score of sadness recognition (disorder-of-happiness group), whereas the medial orbitofrontal region was related to a low score of sadness recognition and a high score of happiness recognition (disorder-of-sadness group). The emotion recognition score in both the happiness and sadness disorder groups was significantly lower than that in the control group (*p* = 0.0009 and *p* = 0.021, respectively). Interestingly, the deficit in happiness recognition was temporary, whereas the deficit in sadness recognition persisted during the chronic phase. Using graph theoretical analysis, we identified structural connectivity between the posterior-prefrontal and medial orbitofrontal regions. When either of these regions was damaged, the tract volume connecting them was significantly reduced (*p* = 0.013). These results indicate that the posterior-prefrontal and medial orbitofrontal regions may be crucial for maintaining a balance between happiness and sadness recognition in humans. Investigating the clinical impact of certain area resections using lesion studies combined with connectivity analysis is a useful neuroimaging method for understanding neural networks.

## Highlights

• Brain areas underlying trade-off relations between emotions were identified.
• Damage to the PPF area reduces accuracy of happiness recognition.
• Damage to the PPF increases accuracy of sadness recognition.
• A similar tendency was observed in orbitofrontal regions for sadness recognition.
• Only a deficit in sadness, but not happiness, persisted in the chronic phase.

## 1 Introduction

Basic human emotions include the following six universal types: anger, disgust, fear, happiness, sadness, and surprise. Large and complex neural networks underlie the recognition of emotions in the human brain, and damage to these networks by various neurological disorders, such as Parkinson’s disease, Huntington’s disease, epilepsy, depression, and dementia, can cause deficits in emotion recognition (Argaud et al., 2018, Dalili et al., 2015, Dogan et al., 2014). Moreover, focal brain damage, including brain tumors, traumatic brain injuries, and stroke, can induce deficits in emotion recognition (Aben et al., 2020, Campanella et al., 2015, Goebel et al., 2018, Nijsse et al., 2019, Yuvaraj et al., 2013).

Each basic emotion is governed by multiple brain regions (Diano et al., 2017, Lindquist et al., 2012, Murphy et al., 2003, Phan et al., 2002). For example, happiness and sadness are related to several brain regions, with happiness being linked to the ventromedial prefrontal cortex, cingulate cortex, insula, and basal ganglia (Phan et al., 2002, Vytal and Hamann, 2010) and sadness to the medial prefrontal cortex (mPFC), anterior cingulate cortex, and orbitofrontal cortex (OFC) (Arias et al., 2020, Murphy et al., 2003, Ouerchefani et al., 2021). Of the several relevant brain regions, the core areas associated with individual emotion types have been established. The core brain region associated with fear is the amygdala (Adolphs et al., 1994, Murphy et al., 2003), and those associated with disgust are the anterior insula, basal ganglia, and ventral prefrontal cortex (Kipps et al., 2007, Suzuki et al., 2006, Vytal and Hamann, 2010). In contrast, the essential brain regions responsible for happiness and sadness recognition are not well understood, although several related brain regions have been implicated (Murphy et al., 2003, Wang et al., 2019). When a given brain region is damaged, representation of a specific emotional type may also be damaged (Motomura et al., 2019, Suzuki et al., 2006). This result might be attributed to the damage to a region containing the networks underlying emotion recognition, possibly the core region for an emotional type. However, some researchers do not consider any specific brain area or circuit to be linked to a basic emotion (Clark-Polner et al., 2017, Lindquist et al., 2012, Touroutoglou et al., 2015).

Previous findings have demonstrated that laterality of emotion recognition is observed on the right side (Gainotti, 2019). For example, facial emotion recognition and recognition of emotional prosody are known to be damaged in the right cerebral hemispheric stroke, specifically in the right frontal lobe (Dara et al., 2013, Harciarek and Heilman, 2009, Harciarek et al., 2006, Kucharska-Pietura et al., 2003, Sheppard et al., 2021). A systematic review comparing the ability to recognize facies, prosodic, and lexical emotional stimuli in stroke between right and left hemispheric lesions also revealed that all categories were significantly impaired in patients with right lesions (Yuvaraj et al., 2013). These previous findings confirm the primacy of the right hemisphere in recognition processing of all emotional experiences across modalities.

In this context, lesion studies, including those involving brain tumor surgery, can provide insights into the changes that might occur in the absence of a specific brain region. In other words, such studies might allow us to identify the brain regions involved in particular emotions (Celeghin et al., 2017). For instance, among several brain regions related to disgust (Diano et al., 2017, Lindquist et al., 2012), the anterior insula and basal ganglia are considered to be the core brain regions, and these regions have been investigated in lesion studies examining Huntington’s disease and Parkinson’s disease (Kipps et al., 2007, Sprengelmeyer et al., 1996, Suzuki et al., 2006). Additionally, these findings have been verified by direct electrical stimulation during brain surgery (Papagno et al., 2016). However, in slow-growing brain tumors, brain function may reorganize preoperatively and may shift from its original functional localization. Although functional reorganization has been observed in language and motor functions (Desmurget et al., 2007, Nakajima et al., 2020), whether it also occurs in emotion recognition, which involves a wide range of brain networks, is not fully understood.

The current cross-sectional study focused on happiness and sadness, respectively representing pleasant and unpleasant emotions, which are universal emotions experienced across diverse geographical regions (Cowen et al., 2021). Indeed, happiness and sadness are closely related and are known to be negatively correlated (Larsen and McGraw, 2011, Watson and Tellegen, 1985). We hypothesized that the essential areas underlying the emotion recognition of happiness and sadness could be identified by examining the influence of focal brain damage on the areas related to these emotions. We investigated the characteristics of emotion recognition deficits using neuroimaging in patients with right cerebral hemispheric brain tumors after surgery.

Several kinds of facial emotional recognition assessments have been developed that are mainly used in Europe and the United States, such as the Reading the Mind in the Eyes test (Baron‐Cohen et al., 2001), Facial expressions of emotion: Stimuli and tests (Young et al., 2002), Pictures of Facial Affect (Ekman and Friesen, 1976), and the University of California, Davis Set of Emotion Expressions (Tracy et al., 2009); however, they are not suitable for Asian countries owing to the differences across nationalities in expression recognition (Matsumoto and Ekman, 1989). The Facial Expression of Emotion task by Matsumoto and Ekman is utilized by Asians as well as individuals from Europe and the United States mainly for research purposes (Biehl et al., 1997, Matsumoto and Ekman, 1988). Here, we designed an expression recognition test for Japanese adults for clinical practice in Japan (Komatsu et al., 2012).

Consequently, we demonstrated the trade-off relationships in related brain regions for happiness and sadness recognition and that the core regions responsible for deficits in happiness recognition could recover, whereas those involved in sadness recognition tended to persist for more than six months after surgery.

## 2 Methods

### 2.1 Participants

Forty-four patients with brain tumors in the right cerebral hemisphere who underwent craniotomies for resection at the Kanazawa University Hospital between August 2013 and March 2018 were enrolled in this study (mean age 52.8 ± 12.9 years). The patient characteristics are summarized in Table 1 and Supplementary Table 1. Generally, postoperative treatment including irradiation and/or chemotherapy was performed for patients with World Health Organization (WHO) classification grade III and IV brain tumors, regardless of the extent of resection, since tumor cells infiltrate beyond the lesions highlighted by magnetic resonance imaging (MRI). We recruited age-matched normal healthy volunteers as a control group (n = 33; mean age 49.9 ± 6.5 years; 12 men and 21 women). There were no significant differences between the two groups in terms of age, sex, handedness, or educational level (Table 1 and Supplementary Table 1). The handedness of all participants was evaluated using the Edinburgh Inventory of Handedness Test (Oldfield, 1971). Informed consent was obtained from all the participants. Additionally, this study was conducted in accordance with the guidelines of the Internal Review Board and approved by the Medical Ethics Committee of Kanazawa University (No. 1797 and 3008).

**Table 1:** Demographic and clinical characteristic of all patients.

Factor	Value		*P*-value
	Right hemispheric brain tumors	Normal healthy volunteers	
Age	52.9 ± 13.1 (31–78)	49.9 ± 6.5 (38–65)	0.48
Sex	Male 24; Female 20	Male 12; Female 21	0.11
Handedness	Right 41; Left 3	Right 33	0.13
Educational level	13.3 ± 2.5	15.0 ± 3.8	0.12
Tumor location	Frontal 30; Temporal 4; Parietal 8; Occipital 2	NA	NA
WHO grade	II 13; III 12; IV 19	NA	NA
Surgical methods	Awake 34; General anesthesia 10	NA	NA
Pre-op tumor volume	36.3 ± 38.5 cc	NA	NA
Extent of resection	88.4 ± 18.9%	NA	NA
Post-op treatment	No 13; Yes 31	NA	NA

NA, not applicable; Wilcoxon test of chi-squared test were utilized. Post-op treatment includes irradiation and/or chemotherapy.

### 2.2 Functional assessment

Emotion recognition ability was assessed using the expression recognition test for adults, the most commonly used basic emotional assessment test in Japan (Komatsu et al., 2012). It consists of 32 photographs of faces with four types of emotional expressions: happiness, sadness, anger, and surprise. Of these, 16 photographs were of male individuals and the rest were of female individuals; there were no duplicate photographs. In the test session, one photograph and five choices (happiness, sadness, anger, surprise, and neutral face) were presented. The participants were asked to select one of the possible descriptions of the presented faces. There were no strict rules for stimulus presentation or inter-stimulus intervals. The patients were informed that the photographs included five types of faces, and they were unaware that neutral faces were not presented. Each correct answer was scored as 1 point, and the total score was calculated by summation (maximum score: 32). The total and subtotal scores for each emotion were recorded. In this study, we focused on happiness and sadness. The assessment was performed at two points: during the first week post-surgery and during the chronic phase (6 months to 1 year post-surgery).

### 2.3 MRI, lesion mapping, and connectivity analysis

All patients underwent structural MRI during the 3-month postoperative period as part of standard care (mean: 2.8 ± 0.6 months). After the initial 6 months, patients underwent MRI every 3 months and every 6 months for WHO grade IV and II/III brain tumors, respectively. Additionally, in principle, the patients underwent diffusion tensor imaging (DTI) 6 months postoperatively. Conventional high-resolution three-dimensional T1-weighted and DTI images were acquired using a 3.0 Tesla MRI scanner (Signa™ Excite HDx 3.0 T; GE Healthcare, Little Chalfont, UK). T1-weighted MRI images were reconstructed according to the Montreal Neurological Institute (MNI) template using the SPM12 software (http://www.fil.ion.ucl.ac.uk/spm/software/spm12/). Images were resliced at a resolution of 1.0 × 1.0 × 1.0 mm. Each MRI image transformed into the MNI space was examined to determine the accuracy of the transformation by comparing anatomical landmarks, such as the sulci and brainstem. If normalization was not accurately performed because of poor-quality MRI or a large resection cavity, the corresponding patients were excluded from the test group. In the current study, one patient was excluded because of insufficient normalization (the patient’s details are not included in Table 1). The resection cavities were reconstructed manually using the MRIcron software (http://www.mccauslandcenter.sc.edu/mricro/mricron/) (Brett et al., 2001). Each reconstruction was initially performed by R.N. and then systematically checked by a neurosurgeon (M.K.). We calculated the extent of resection by noting the hyperintense areas on T2-weighted images for grade II/III tumors and the contrast-enhanced areas for grade IV tumors.

For the DTI analysis, 30 diffusion sampling directions were acquired. The b-value was 1000 s/mm^2^. The in-plane resolution was 0.86 mm, and the slice thickness was 2.5 mm. The diffusion data were reconstructed in the MNI space using q-space diffeomorphic reconstruction to obtain the spin distribution function (Yeh and Tseng, 2011, Yeh et al., 2010). A diffusion sampling length ratio of 1.25 was used. The output resolution was 1 mm and isotropic. The analysis was conducted using DSI Studio (http://dsi-studio.labsolver.org). We used a deterministic algorithm to prevent false-positive errors; false-negative errors could be tolerated to some extent since we aimed to investigate whether connections existed between the medial orbitofrontal (mOF) and posterior-prefrontal (PPF) regions (Sarwar et al., 2019). The tractography was generated using an anisotropy threshold of 0.18 and an angular threshold of 60°. The step size was randomly selected between 0.5 and 1.5 voxels. Regions of interest (ROIs) were placed on the lesions identified in the imaging analysis, as described below. A total of 1000 streamlines were generated for each patient. The tract volume was automatically computed using DSI Studio. To calculate resected volume of the analyzed tract, resection cavities were overlayed on the preoperative tract in each patient. The overlayed volume (voxels) was calculated automatically on MRIcron software (http://www.mccauslandcenter.sc.edu/mricro/mricron/). In addition, we used a group average template constructed from 1021 subjects obtained from the Human Connectome Project. A multishell diffusion scheme was used, and the b-values were 990, 1985, and 2980 s/mm^2^, each with 90 diffusion sampling directions. The value of both the in-plane resolution and slice thickness was 1.25 mm. The protocol for the reconstruction of the diffusion data to the MNI space and tracking parameters was as described above. A diffusion sampling length ratio of 2.5 was used, and the output resolution was 1 mm. Restricted diffusion was quantified using diffusion-restricted imaging. ROIs were used for brain parcellation, and the connectivity matrix was calculated using the number of connecting tracks. All analyses were performed using DSI Studio.

### 2.4 Statistical analysis

Voxel-based lesion-symptom mapping (VLSM) was performed to investigate the relationship between the score of expression recognition and the location of the surgical resection cavity (Bates et al., 2003). The dependent variables were standardized residuals for the happiness and sadness recognition scores. The NPM software in the MRIcron package was used for statistical analyses of neuroimaging data and continuous (or binary) data. To minimize possible outlier effects, analyses were performed only on voxels corresponding to brain damage from at least four individuals, as previously reported (Aiello et al., 2012). Parametric *t*-tests were performed on continuous data to compute statistical maps. To control for false-positive errors, a false discovery rate correlation was applied, with a threshold of *p* = 0.05. Significant differences between the test scores were identified and presented visually as Z-scores on the MNI template.

To obtain behavioral data, scores from the expression recognition test in the patient group were collected at two points: during the first postoperative week and during the chronic phase. All raw test scores of the behavioral data were transformed into standardized residuals. Age, educational level, and sex were entered as predictors using a statistical analysis software (SPSS, version 27.0; IBM Corp., Armonk, NY, USA). This approach is widely accepted and commonly used to analyze neuropsychological data when several covariates are out-of-control factors (Godefroy et al., 2014, Herbet et al., 2014). To show the patients’ responses to each emotional faces, we calculated the ratio of the total number of each response of happiness, sadness, anger, surprise, and neutral face to the total trial number of each emotional faces. As the evaluation index, positive predictive value (PPV) of responses for each emotional faces were calculated with the following equation: number of true positives / (number of true positives + false positives). Nonparametric tests, including the Steel test, were applied because the data did not follow a normal distribution. A statistical analysis software (JMP, version 14.0.0; SAS Institute, Inc., Cary, NC, USA) was used to perform all statistical analyses, except for the calculation of standardized residuals, as described above.

## 3 Results

### 3.1 Anatomical data

**Fig. 1:**
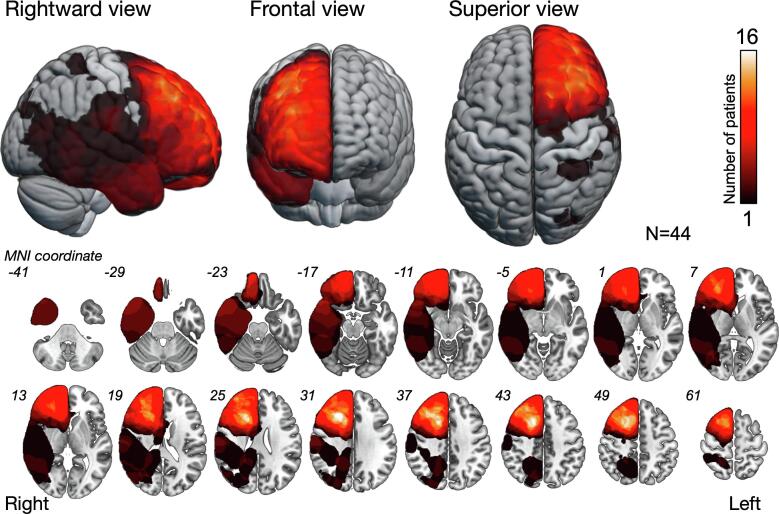
Overlap map of resection cavities. The overlap map of resection cavities shows that the greatest overlap was in the deep part of the middle frontal gyrus (white regions, n = 16).

Resection cavities were located in the frontal, temporal, parietal, and occipital lobes in 30, 4, 8, and 2 patients, respectively. Fig. 1 illustrates the overlap map of all the resection cavities (n = 44). The greatest overlap of the resection cavities was observed in the deep part of the middle frontal gyrus (n = 16).

### 3.2 VLSm

**Fig. 2:**
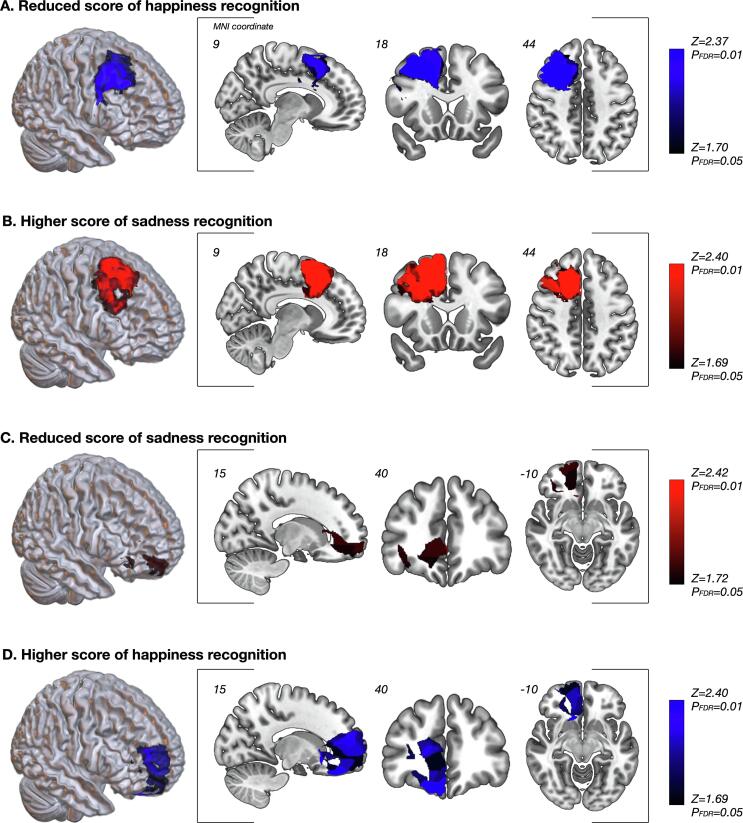
Results of the Voxel-based lesion-symptom mapping. Fig. 2A and B show spatial locations related with reduced score in happiness recognition and higher score in sadness recognition, respectively. The statistical map shows voxels that were significant according to the false discovery rate-controlled threshold (*p* = 0.05; *z* = 1.70 for 2A, *z* = 1.69 for 2B, respectively). Significant regions presented in 2A and 2B largely overlapped. Similarly, regions shown in 2C mostly overlapped that of 2D. The statistical map shows the voxels that were significant according to the false discovery rate-controlled threshold (*p* = 0.05; *z* = 1.72 for 2C, and *z* = 1.69 for 2D).

VLSM was performed to identify the brain regions that were significantly related to deficits in happiness and sadness recognition. During the acute phase, the largest cluster of significant voxels sensitive to a low score in happiness recognition was located in the superior to middle frontal gyri and medial frontal regions (cluster size, 39,865; Z_max_ = 3.89, Fig. 2A). In contrast, a significant cluster underlying higher sensitivity in sadness recognition was found in the deep part of the dorsolateral to medial prefrontal cortices (cluster size, 50,083; Z_max_ = 4.06, Fig. 2B). Moreover, voxels with significantly reduced score in sadness recognition were located in the anterior part of the cingulate cortex to the orbitofrontal region (cluster size, 14,810; Z_max_ = 3.10, Fig. 2C). In contrast, a significant cluster for high score in happiness recognition was identified in the anterior part of the prefrontal regions, including the orbitofrontal area (cluster size, 44,380; Z_max_ = 3.41, Fig. 2D).

**Supplementary figure 1:**
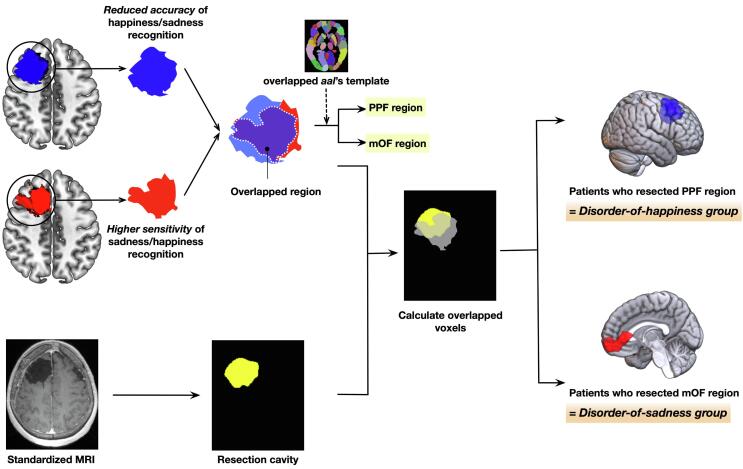


Interestingly, the largest parts of the following region pairs overlapped: regions for low score in happiness recognition and high score in sadness recognition (Fig. 2A and B) and regions for low score in sadness recognition and high score in happiness recognition (Fig. 2C and D). Based on these results, we further investigated the characteristics of emotion recognition in the patient group (Supplementary Fig. 1). The two regions presented in Fig. 2A and B, the regions sensitive to a low score in happiness recognition and a high score in sadness recognition, including the posterior superior to middle frontal gyri and the medial part of the premotor and prefrontal regions, overlapped in an area of 203,345 voxels and were designated as the PPF region. Similarly, as shown in Fig. 2C and D, the regions reflecting a low score in sadness recognition and a high score in happiness recognition, mainly including the medial part of the OFC and the anterior part of the cingulate cortex, overlapped in an area of 101,871 voxels and were designated as the mOF region. Subsequently, the resection cavity and PPF/mOF regions of each patient were superimposed, and overlapping voxels were automatically calculated. Patients with resected PPF or mOF regions were grouped into disorder-of-happiness or disorder-of-sadness groups, respectively. Notably, two patients who underwent resection of the PPF and part of the mOF were grouped into the disorder-of-happiness group, since the central region of the tumor resided in the PPF. Consequently, the number of cases (n) in the disorder-of-happiness and disorder-of-sadness groups was 19 and 7, respectively. In 18 patients, neither the PPF nor mOF regions were resected (“other patients” group).

### 3.3 Behavioral data

**Fig. 3:**
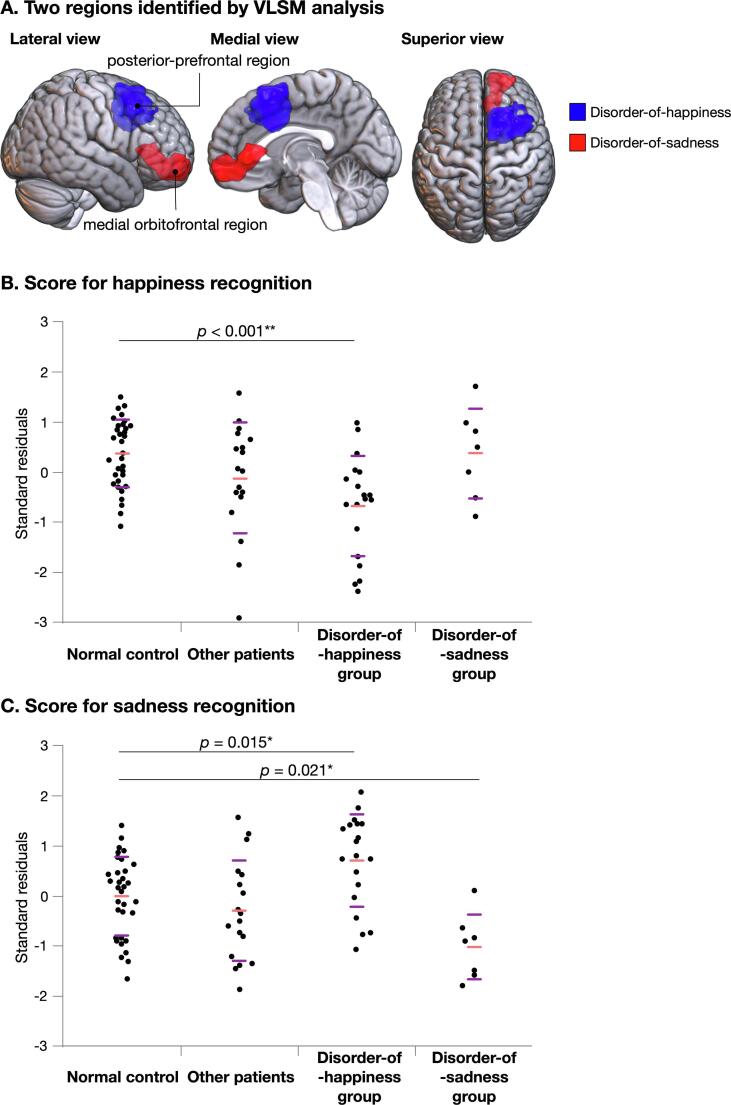
Score for emotion recognition. Two regions were identified using the Voxel-based lesion-symptom mapping as related to emotion recognition deficit (A). Score for emotion recognition was compared among 4-groups using Steel test: Disorder-of-happiness group who had their posterior-prefrontal region resected, and disorder-of-sadness group who had their medial orbitofrontal region resected, other patients who had neither the posterior-prefrontal region nor the medial orbitofrontal region resected, and normal healthy controls. The score for happiness recognition was significantly lower in disorder-of-happiness group than the normal controls (B). When score of sadness was compared with normal control, disorder-of-sadness group showed significant reduced score, while disorder-of-happiness group showed significantly higher score (C). Purple line in 3B and 3C, standard deviation; light red line in 3B and 3C, mean; *p < 0.05, ^**^p < 0.001. (For interpretation of the references to colour in this figure legend, the reader is referred to the web version of this article.)

**Fig. 4:**
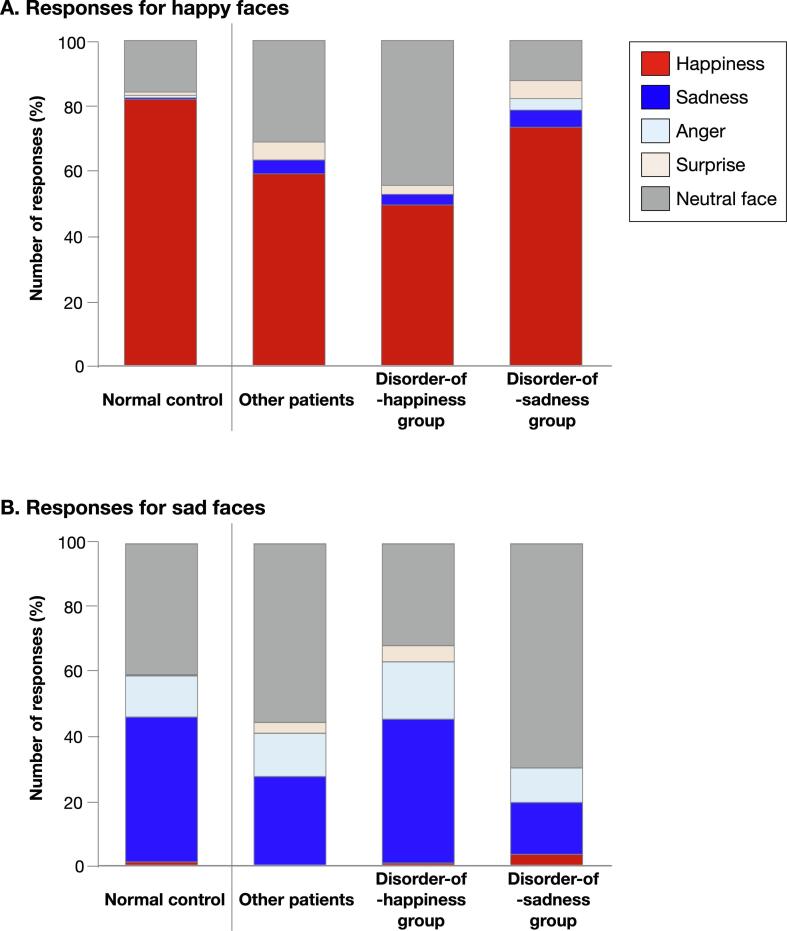
Details of patient response. 4A: Responses of “happiness” for happy face in disorder-of-happiness group was lower compared with normal control group, instead responses of “neutral face” were much than other groups. Response pattern of disorder-of-sadness group was similar to that of normal control group. 4B: Similarly, responses for “sadness” in disorder-of-sadness group was lower than normal control group, while responses of “neutral face” were much than other groups. Response pattern of disorder-of-happiness group was similar tendency to that of normal control group.

Scores were compared between the groups with right frontal brain tumors (namely the disorder-of-happiness, disorder-of-sadness, and “other patients” groups; Fig. 3A) and the control groups. There were no significant differences in age, sex, educational level, and MRI scanning time since surgery among four groups (Supplementary Table 1). We found the following findings through the Steel analysis: the score for happiness recognition in the disorder-of-happiness group was significantly lower than that in the controls (*p* < 0.001, Z = −3.62, lower/upper confidence limit = −1.69/−0.31, effect size [r] = −0.50). The score for happiness recognition in the disorder-of-sadness and “other patients” groups was not significantly different from that in the control group (*p* = 1.00 [Z = −0.036, lower/upper confidence limit = −0.91/0.91, r = −0.01] and *p* = 0.30 [Z = −1.57, lower/upper confidence limit = −1.09/0.25, r = −0.22], respectively; Fig. 3B). Similarly, the score for sadness recognition in the disorder-of-sadness group was significantly lower than that in the control group (*p* = 0.021, Z = −2.69, lower/upper confidence limit = −1.85/-0.14, r = −0.43, Fig. 3C) and was significantly higher in the disorder-of-happiness group than in the control group (*p* = 0.015, Z = 2.80, lower/upper confidence limit = 0.12/1.36, r = 0.39). Patient responses are shown in Fig. 4 (Supplementary Table 2. See also Supplementary Fig. 2 for other emotional faces.). Among the incorrect responses for happiness in the disorder-of-happiness group, neutral faces were the most common (44.7%). This finding suggests that the patients confused happy faces with neutral faces. Correct responses of disorder-of-happiness group for happy faces was the lowest among four groups (PPV = 0.49, Supplementary Table 2). In contrast, 73.2% of the disorder-of-sadness group associated happiness with happy faces, an observation similar to that observed in the normal control group (81.8%). In total, 16.1% of the disorder-of-sadness group, representing the lowest percentage among all groups, responded by linking sadness to sad faces (PPV = 0.16). The proportion of patients who associated sad faces with sadness in the disorder-of-happiness group was almost identical to that in the normal control group (44.7% and 45.1%, respectively). Importantly, disorder-of-happiness group does not have increased tendency to recognize any emotion as sadness. It is same for happiness recognition in disorder-of-sadness group (Supplementary Fig. 2).

**Fig. 5:**
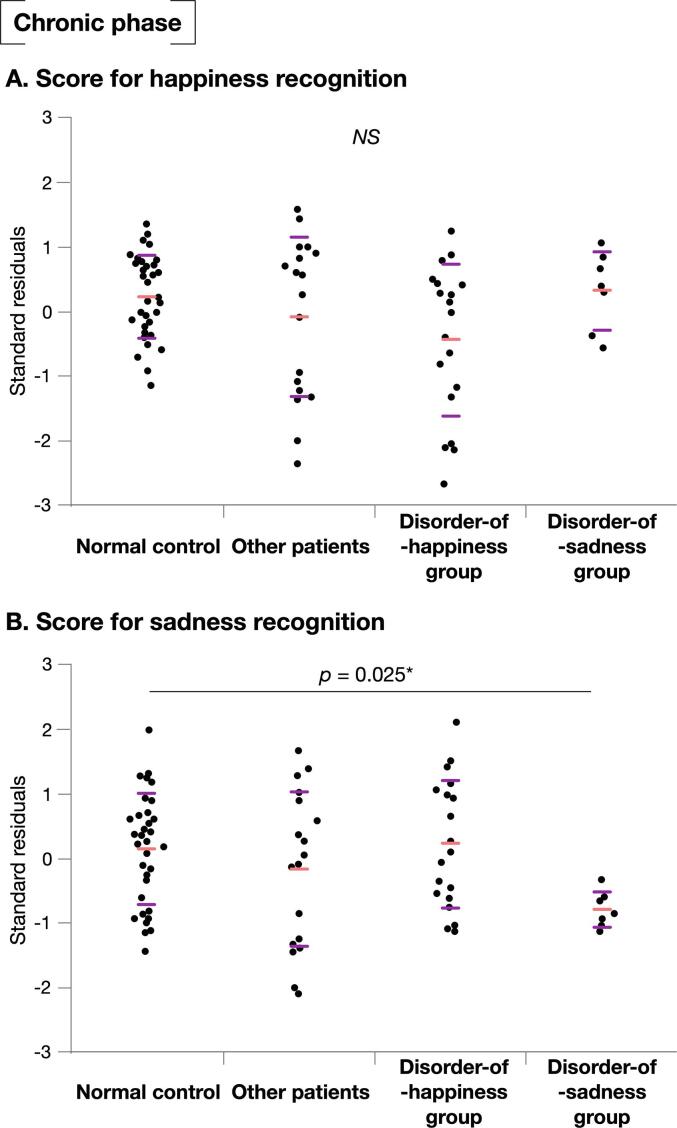
Score for emotion recognition at the chronic phase. Every patient was performed test of emotion recognition at least twice; postop 1 week and chronic phase, within postop 6 months to 1 year. Emotion recognition was compared among 4 groups using Steel test. As for score of happiness recognition, all group showed similar tendency, and there were no significant differences among the groups (A). Score for sadness recognition in the disorder-of-sadness group was still significantly lower than the control group (B). Purple line, standard deviation; light red line, mean; *p < 0.05. (For interpretation of the references to colour in this figure legend, the reader is referred to the web version of this article.)

In the chronic phase, there were no significant differences between the groups with regard to happiness (Fig. 5A). However, the score for sadness responses in the disorder-of-sadness group was still significantly lower than that in the control group (*p* = 0.025, Z = -2.63, lower/upper confidence limit = -1.64/-0.050, r = -0.42, Fig. 5B). Notably, the scores for happiness and sadness in other patients showed similar tendencies, with no significant difference compared with the control group.

### 3.4 Connectivity analysis

**Fig. 6:**
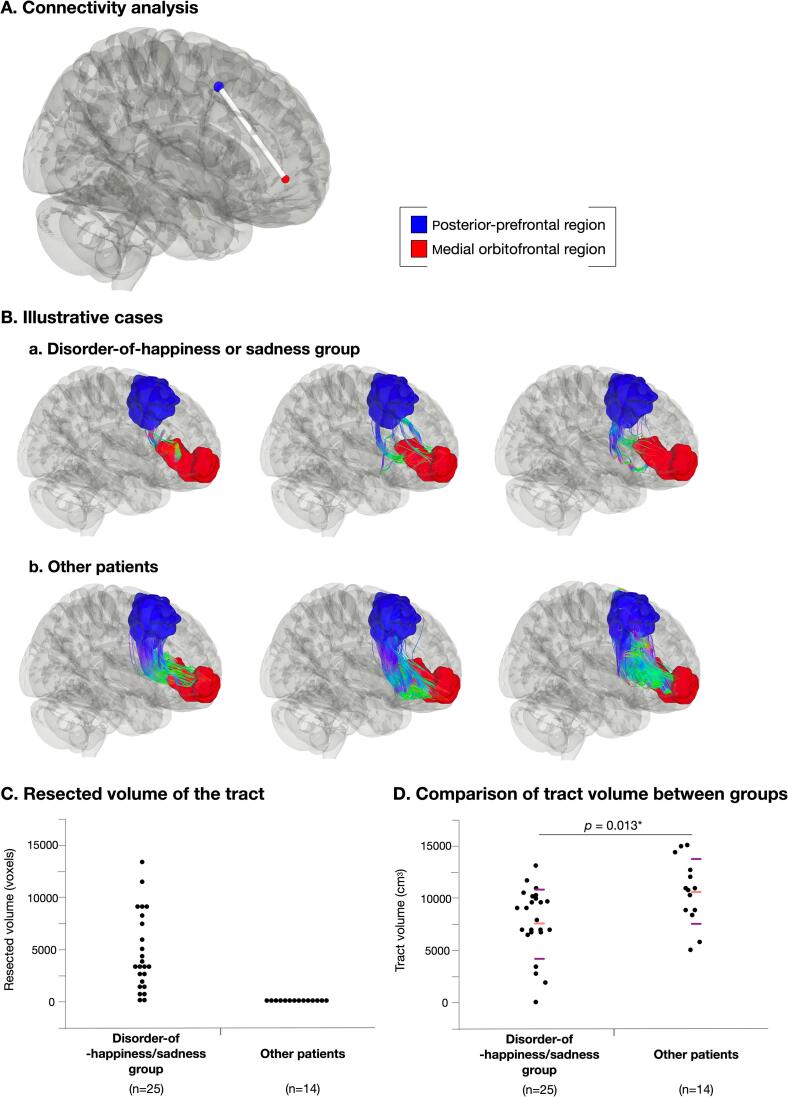
Structural connectivity between posterior-prefrontal region (blue) and medial orbitofrontal region (red) were found via connectivity matrix and graph theoretical analysis using group average template (A). Illustrative cases of “disorder-of-happiness or sadness group” (B-a), and “other patients’ group”, which means neither region was resected (B-b) are shown in the middle low. Resected volume of the tract was calculated in each patient (C). Then, tract volume between these two groups were compared using Wilcoxon test. Results indicates that tract volume of disorder-of-happiness or sadness group were smaller significantly than that of other patients’ group (D). *p < 0.05. (For interpretation of the references to colour in this figure legend, the reader is referred to the web version of this article.)

To identify the structural connectivity between the PPF and mOF, we first analyzed the connectivity matrix and employed a graph theoretical approach using a group average template and found structural connectivity between the two regions (Fig. 6A). The DTI of patient groups was compared between the disorder-of-happiness/sadness group, namely patients with any of the regions removed (n = 25), and other patients, namely patients with none of the regions removed (n = 14); however, postoperative data were not available for five patients. Structural connectivity between the PPF and mOF regions was analyzed and could be drawn in all patients, except for one case in the disorder-of-happiness/sadness group. The shape of the streamline indicates a poor tract volume caused by partial resection of the white matter in the disorder-of-happiness/sadness group and a large tract volume in the “other patients” group (patients with none of the regions resected) (Fig. 6B). Resected volume of the tract was shown in Fig. 6C. Tract volume was compared between groups using the Wilcoxon test; the tract volume in the disorder-of-happiness/sadness group was significantly smaller than that in the “other patients” group (Fig. 6D, *p* = 0.013, Z = 2.47, lower/upper confidence limit = −2752.5/−5149.0, r = 0.40).

## 4 Discussion

**Fig. 7:**
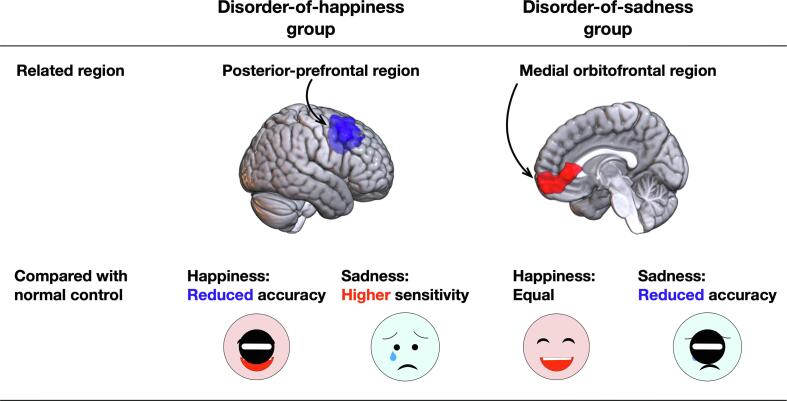
Summary of the current study.

This study investigated the characteristics of emotion recognition in patients with facial expression deficits following brain tumor surgery in the right cerebral hemisphere. We found that damage to the PPF region was significantly related to a low score in happiness recognition and high score in sadness recognition. In comparison, damage to the mOF region was significantly associated with a low score in sadness recognition and high score in happiness recognition, resulting in a trade-off relationship (Fig. 7). The score of emotion recognition in both the happiness and sadness disorder groups was significantly lower than that in the control group. Additionally, postoperative recovery differed depending on the emotion affected: patients in the disorder-of-happiness group recovered before the chronic phase, whereas those in the disorder-of-sadness group hardly recovered, even in the chronic phase. In the current study, we investigated the influence of focal brain damage on the recognition of happiness and sadness. We showed that the identified regions may be essential brain structures for facial emotional recognition networks.

Previous studies have demonstrated that several brain regions are related to happiness recognition, such as the medial prefrontal and dorsolateral prefrontal cortices, insula, and basal ganglia, in both healthy participants and patients with psychiatric disease, although the critical mechanism underlying emotion recognition is not well understood (Diano et al., 2017, Haller et al., 2018, Keener et al., 2012, Phan et al., 2002, Surguladze et al., 2010). We considered the mPFC to be an important area in happy facial expression recognition because our results suggest that happiness, but not sadness, is impaired by damage to the mPFC. Our interpretation seems to be consistent, at least partially, with the results of previous studies. Functional imaging studies of healthy controls have indicated that the prefrontal cortex, including the middle frontal gyrus, may be involved in identifying a specific emotion from multiple emotions for any type of basic emotion (Diano et al., 2017, Saarimäki et al., 2016). Moreover, another functional imaging studies of healthy controls revealed that mPFC activation is related to positive mood during cognitive task (Subramaniam and Vinogradov, 2013). Thus, although the mPFC is not the only region associated with happiness, it is one of the central regions representing positive emotions, including happiness.

Regarding sadness recognition, previously published neuroimaging and *meta*-analysis studies for both healthy and brain-damaged participants have implicated related brain regions, such as the right orbitofrontal and ventromedial prefrontal cortices, including the cingulate cortex (Arias et al., 2020, Diano et al., 2017, Murphy et al., 2003, Ouerchefani et al., 2021, Vytal and Hamann, 2010). However, as with happiness recognition, several brain regions are known to be involved in sadness recognition, and the core region is not well understood (Murphy et al., 2003, Wang et al., 2019). In the present study, focal brain damage to the OFC caused a deficit in processing sad facial expressions, suggesting that the OFC is one of the key areas of the network involved in sadness recognition. Indeed, the OFC is part of the limbic system and is related to emotion recognition and emotional self-regulation—the process of amplifying, attenuating, or maintaining an emotion—together with the anterior cingulate cortex, dorsolateral prefrontal cortex, and amygdala (Arias et al., 2020, Davidson et al., 2000, Lévesque et al., 2003). The amygdala-orbitofrontal functional connectivity is involved in voluntary sadness suppression and plays a role in controlling sad emotions (Versace et al., 2010). However, some researchers have pointed out that brain regions related to sadness are labile and lack consistency (Arias et al., 2020, Eugène et al., 2003, Wager et al., 2015).

Owing to the generally low percentage of correct answers for sadness in the current study, the low scores in the disorder-of-sadness group might be considered to simply reflect the task difficulty. However, we believe that the disorder-of-sadness group selectively exhibited a decrease in the recognition of sadness because of the following two reasons: only this group showed a lower score of sadness recognition than the normal control group, and their score of happiness recognition was nearly normal.

In this study, regions related to happiness and sadness deficits demonstrated a trade-off relationship. Observations similar to those of the current study have been previously published in only few reports (Bomfim et al., 2019, Keding and Herringa, 2016). In line with the results of our study, a recent neuroimaging study revealed that patients with major depression showed a high accuracy in recognizing sad and angry faces and a low accuracy in happiness recognition (Bomfim et al., 2019, Nyquist and Luebbe, 2020). In the human brain, the physical disruption of certain brain areas may result in other regional increases in functional connectivity, namely, hyperconnectivity (Hillary and Grafman, 2017). In our patient group, loss of happiness recognition (loss of emotion discriminative ability) resulted in hypersensitivity to sadness (further loss in emotion discriminative ability). Similarly, disruption of a network resulting in hyperactivity of another region that is structurally and functionally connected to the disrupted network has been previously observed in multiple studies, including those examining traumatic brain injury, and brain tumor (Bartolomei et al., 2006, Hillary et al., 2014, Johnson et al., 2012). In the current study, we speculated that damage to either the mOF or PPF region may disturb the emotional balance between happiness and sadness. We then performed connectivity analysis as second level analysis to investigate the structural mechanism of the trade-off relationships between these two. Interestingly, we identified brain regions related to happiness and sadness disorders that were structurally connected. In addition, damage to either of these regions resulted in decreased anatomical connectivity. Functional connectivity between these two regions, namely the PPF and mOF, has been observed in studies examining psychiatric diseases (Feldstein Ewing et al., 2017, Frodl et al., 2010). Similarly, in the present study, structural connections between the orbitofrontal or ventromedial prefrontal cortex and the prefrontal cortex were observed (Jackson et al., 2020). Indeed, the cingulum bundle is known to play an important role in emotion regression, and its structural abnormality can cause psychiatric disorders with abnormal emotion regulation (Heilbronner and Haber, 2014, Versace et al., 2019). These findings provide anatomical evidence to support the notion that damage to the connectivity between the PPF and mOF regions caused by either of these two regions breaks the balance between happiness and sadness. However, since the decrease in connectivity between the PPF and mOF regions were consequence of the resection of one or the other, it is not clear whether the same thing would occur in cases only the connectivity was damaged while both regions were preserved. Another possibility is that patients with damage to PPF show higher accuracy on sadness recognition because they are less likely to have damage in region that is important for sadness recognition. The same is true for happiness recognition of patients with damage to the mOF. We will continue to work on elucidating the trade-off relationships between different emotions.

Previous studies reported that most neuropsychological deficits, including those in emotion recognition and mentalizing, could recover within three postoperative months, despite a temporary decline (Herbet et al., 2013, Nakajima et al., 2017, Nakajima et al., 2018). In this study, we demonstrated for the first time that the emotion recovery process can differ depending on the emotion. Further research is required to investigate the cause of these differences.

This study had some limitations. First, the available behavioral data in the current study are limited because the neuropsychological examination was performed in clinical practice, focusing on functions related to a return to social life. Specifically, this study was limited to facial emotional recognition. In reality, it is ideal to use several types of assessments of emotional function or subjective depression, which may influence emotion recognition. Second, the results on the correlation between neural circuits and emotional function lack consistency and may vary across different studies, despite the investigation of the same emotional state (Eugène et al., 2003). We believe that this inconsistency results from the high complexity of brain networks interconnecting specific regions that govern emotions. In other words, a specific brain region may be related to a particular emotion, however, not exclusively. It means that while the PPF and mOF relate to happiness and sadness recognition, but other regions which we could not sufficiently explore also involved in happiness and sadness recognition. Actually, the VLSM has the limitation of not being able to study areas other than the lesion of the patient group. In this context, our results are significant because we could causally correlate defects in the accuracy of emotion recognition in the absence of specific brain structures. Finally, the concept of a basic emotion itself is a matter of debate (Hutto et al., 2018). Some researchers have argued that each emotion is governed by different brain networks (Clark-Polner et al., 2017, Touroutoglou et al., 2015). Considering all these viewpoints and findings, we aim to continue investigating this topic in subsequent cases in combination with other analytic methods in the future.

## 5 Conclusions

The right PPF and mOF regions were related to emotional recognition of facial expressions for happiness and sadness respectively, and damage of either region can disturb the balance between these two. Although the deficit in happiness recognition recovered before the chronic phase, the deficit in sadness recognition persisted during the chronic phase. Our results, which show the influence of focal brain damage on the recognition of happiness and sadness, may therefore be useful for understanding an essential aspect of the facial emotional recognition network.

## Figures and Tables

**Supplementary figure 2:**