# Supplementary material for: Posterior-prefrontal and medial orbitofrontal regions play crucial roles in happiness and sadness recognition
Source: Neuroimage Clin. 2022 Jun 2;35:103072. doi: 10.1016/j.nicl.2022.103072 (PMC9192961; doi:10.1016/j.nicl.2022.103072)
Supplement: Supplementary data 1 [file mmc1.docx]

**Supplementary Table 1**. Comparison of demographic and clinical factors among four groups

| **Factor** | **Value** | | | | ***P*-value** |
| --- | --- | --- | --- | --- | --- |
|  | **Normal controls** | **Other patients** | **Disorder of happiness group** | **Disorder of sadness group** |  |
| Age | 49.9±6.5 | 55.7±13.1 | 50.1±12.5 | 53.4±14.9 | 0.38 |
| Sex; male/Female | 12/21 | 10/8 | 12/7 | 2/5 | 0.17 |
| Educational level (year) | 15.0±3.8 | 12.6±2.7 | 14.1±2.2 | 13.1±2.3 | 0.078 |
| MRI timing after surgery (months) | NA | 2.7±0.7 | 2.8±0.5 | 3.0±0.8 | 0.46 |

In statistical analysis, Chi-square test and Wilcoxon test with Bonferroni correction were utilized. NA, not applicable.

**Supplementary Table 2**. Confusion matrix for patient responses

|  |  | Presented faces | | | |
| --- | --- | --- | --- | --- | --- |
|  |  | Happiness | Sadness | Anger | Surprise |
| ***Normal controls*** | | | | | |
| Patients’ responses | Happiness | 216 | 3 | 23 | 3 |
|  | Sadness | 1 | 119 | 26 | 5 |
|  | Anger | 2 | 33 | 148 | 3 |
|  | Surprise | 3 | 1 | 6 | 192 |
|  | Neutral face | 42 | 108 | 61 | 61 |
|  | PPV | 0.82 | 0.45 | 0.56 | 0.73 |
| ***Other patients*** | | | | | |
| Patients’ responses | Happiness | 85 | 0 | 19 | 2 |
|  | Sadness | 6 | 40 | 12 | 1 |
|  | Anger | 0 | 19 | 59 | 0 |
|  | Surprise | 8 | 5 | 7 | 105 |
|  | Neutral face | 45 | 80 | 47 | 36 |
|  | PPV | 0.59 | 0.27 | 0.41 | 0.73 |
| ***Disorder of happiness group*** | | | | | |
| Patients’ responses | Happiness | 75 | 1 | 8 | 6 |
|  | Sadness | 5 | 68 | 14 | 4 |
|  | Anger | 0 | 27 | 83 | 4 |
|  | Surprise | 4 | 8 | 15 | 111 |
|  | Neutral face | 68 | 48 | 32 | 27 |
|  | PPV | 0.49 | 0.45 | 0.55 | 0.73 |
| ***Disorder of sadness group*** | | | | | |
| Patients’ responses | Happiness | 41 | 2 | 6 | 3 |
|  | Sadness | 3 | 9 | 4 | 4 |
|  | Anger | 2 | 6 | 21 | 2 |
|  | Surprise | 3 | 0 | 5 | 31 |
|  | Neutral face | 7 | 39 | 20 | 16 |
|  | PPV | 0.73 | 0.16 | 0.38 | 0.55 |

The positive predictive values (PPV) were calculated as follows; number of true positives / (number of true positives + false positives).
